# Multivariable regression analysis of list experiment data on abortion: results from a large, randomly-selected population based study in Liberia

**DOI:** 10.1186/s12963-017-0157-x

**Published:** 2017-12-21

**Authors:** Heidi Moseson, Caitlin Gerdts, Christine Dehlendorf, Robert A. Hiatt, Eric Vittinghoff

**Affiliations:** 1 Department of Epidemiology & Biostatistics, University of California, San Francisco, USA; 2 Ibis Reproductive Health, 1330 Broadway St, Suite 1100, Oakland, CA 94612 USA; 3 Department of Family & Community Medicine, University of California, San Francisco, USA

**Keywords:** Abortion, List experiment, Item count technique, Multivariable regression analysis, Design effect, Liberia, Family planning, Methods

## Abstract

**Background:**

The list experiment is a promising measurement tool for eliciting truthful responses to stigmatized or sensitive health behaviors. However, investigators may be hesitant to adopt the method due to previously untestable assumptions and the perceived inability to conduct multivariable analysis. With a recently developed statistical test that can detect the presence of a design effect – the absence of which is a central assumption of the list experiment method – we sought to test the validity of a list experiment conducted on self-reported abortion in Liberia. We also aim to introduce recently developed multivariable regression estimators for the analysis of list experiment data, to explore relationships between respondent characteristics and having had an abortion – an important component of understanding the experiences of women who have abortions.

**Methods:**

To test the null hypothesis of no design effect in the Liberian list experiment data, we calculated the percentage of each respondent “type,” characterized by response to the control items, and compared these percentages across treatment and control groups with a Bonferroni-adjusted alpha criterion. We then implemented two least squares and two maximum likelihood models (four total), each representing different bias-variance trade-offs, to estimate the association between respondent characteristics and abortion.

**Results:**

We find no clear evidence of a design effect in list experiment data from Liberia (*p* = 0.18), affirming the first key assumption of the method. Multivariable analyses suggest a negative association between education and history of abortion. The retrospective nature of measuring lifetime experience of abortion, however, complicates interpretation of results, as the timing and safety of a respondent’s abortion may have influenced her ability to pursue an education.

**Conclusion:**

Our work demonstrates that multivariable analyses, as well as statistical testing of a key design assumption, are possible with list experiment data, although with important limitations when considering lifetime measures. We outline how to implement this methodology with list experiment data in future research.

**Electronic supplementary material:**

The online version of this article (10.1186/s12963-017-0157-x) contains supplementary material, which is available to authorized users.

## Background

The incidence and prevalence of abortion are notoriously difficult to measure. [1–5] Women have reservations about reporting abortion experiences due to legal concerns, and to worries about privacy and stigma, resulting in under-reporting in direct surveys. [2, 4] Inaccurate measurement of the incidence and prevalence of abortion limits the effectiveness of policy and program planning.

The list experiment is a promising measurement tool for eliciting truthful responses to stigmatized or sensitive health behaviors that has recently been applied to the measurement of abortion. [6–8] Originating in the 1980s, the list experiment is frequently used in the political science and economics literatures, though rarely – if at all – in public health and epidemiology. The method, described in detail elsewhere, [9–12] is designed to protect the confidentiality of a respondent’s answer to a sensitive question. In its simplest form, the list experiment works by dividing a study sample into two randomly selected groups. In the control group, the respondent is shown a list of non-sensitive beliefs or experiences, and then is prompted to report how many of the items are true for him or her, but not to specify which ones. The treatment group is shown the same list of non-sensitive items, but a sensitive item – e.g., abortion – is added. The treatment group participants are similarly asked to report how many of the items are true for them, but not which ones. The difference in means between the numbers of items reported for the treatment list versus the control list is typically used as an estimate of the population proportion that has experienced the sensitive item (e.g., abortion). The method relies on two core assumptions: first, the assumption of no design effect – that participants do not change their response to the control items based on the presence or absence of the treatment item; and second, that participants give a truthful answer to the sensitive item. [13]

In the first list experiment on abortion, estimates suggested that 32% (95% CI: 0.29, 0.34) of women in Liberia had ever had an abortion – an estimate five times greater than the only previous representative estimate of abortion in Liberia. [6] A list experiment to measure lifetime history of abortion in the United States estimated that 22% of women in the sample had ever had an abortion – 4% higher than estimates resulting from direct questioning. [7] At least half a dozen other list experiments to measure abortion are now underway around the world. [8]

However, some family planning researchers have been hesitant to adopt the method due to seemingly untestable key assumptions, most notably the absence of a design effect. In the context of the list experiment, there is a design effect when a respondent reports a different number of control items as true for her depending on whether or not the sensitive item is included in the list. [14] For instance, if a respondent were read the control version of a list and would truthfully report that two of three control items were true for her, but, if read the version of the list with the sensitive item added, would instead report that only one of the control items were true for her (to reduce the chance of the enumerator guessing that she might have experienced the sensitive item), then a design effect is present, and list experiment estimates will be biased.

In addition to concerns about the ability to test for a design effect, multivariable analyses to explore factors associated with history or incidence of abortion using the list experiment have not been done. While stratum-specific estimates are straightforward to calculate with list experiment data, this becomes untenable as the number of covariates that must be adjusted for increases, in addition to being statistically inefficient. [14] However, recent methodological work from other disciplines has introduced a statistical test for the assumption of no design effect, as well as two multivariable regression estimators for use with list experiment data. [13–15] In this paper, we applied these methods and tested the validity of the Liberian list experiment on abortion with this recently developed design effect test. We also conducted a multivariable analysis of the Liberian list experiment data, to demonstrate how relationships between respondent characteristics and having had an abortion can be conducted with these newly developed estimators for list experiment data.

## Methods

### Study sample

Using geographic information system data on spatially defined enumeration areas (EAs) developed in the 2008 National Liberian Census, we used an R script to randomly select 176 EAs in Bomi (primarily rural) and Montserrado (urban) counties in Liberia with probability proportional to size. Within these EAs, women between the ages of 15 and 49 years were randomly selected within the approximately 3500 households that were themselves selected based on enumerator ordering from a random start. All women were recruited in June and July of 2013, and all participants gave verbal confirmation of informed consent. More details on study sampling and recruitment can be found elsewhere [6].

### Ethics

This research was approved by the ethical review board of the Liberian Ministry of Health, and by the Committee on Human Research at the University of California, San Francisco.

### List experiment design

**Fig. 1:**
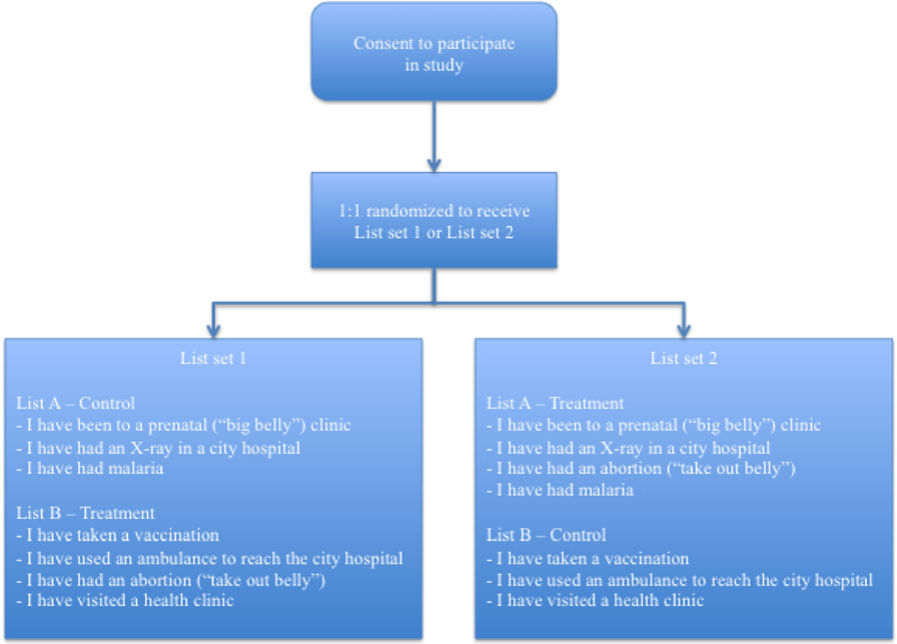
Diagram of double list experiment administration

To measure lifetime prevalence of abortion, we used a double list experiment. [9, 10] In this double list experiment, the study sample was randomly split into two groups. Both groups received two lists of non-sensitive health experiences (List A and List B). Abortion was randomly added to either List A or List B, and the other list was kept in its original form. Both groups received both lists, with only one of the two lists containing abortion, and thus, each group served as the “control” for the other (see Fig. 1). For each list, the respondent provided the total *number* of items that she had experienced, not which ones. The numbers provided for each list were summed across respondents and averaged by list. The averages for the control versions of List A and List B were then subtracted from the treatment versions of List A and List B to generate an estimate of the population proportion that had ever had an abortion. These two estimated abortion prevalences (one from the difference between the treatment and control versions of List A, and another from the difference between treatment and control versions of List B) were then averaged to arrive at a final estimate. The specific lists received by each group read as follows:

List A: Here is a list of three things that some people have done. Please listen to them and tell me HOW MANY you have experienced. Do not tell me which ones, just tell me how many. Here are the three things:

1. I have been to a prenatal (“big belly”) clinic.
2. I have had an X-ray in a city hospital.
3. I have had malaria.

Now, how MANY of these have you experienced? None, one, two, or all?

List B: Now I am going to read you another list. Please listen to all of the things and then tell me HOW MANY you have done. Again, not which ones, just how many.

1. I have taken a vaccination.
2. I have used an ambulance to reach the city hospital.
3. I have visited a health clinic.

Now, how MANY of these are true for you? None, one, two, or all? [6].

Abortion was randomly added to either List A or List B for each respondent, in the following form: “4. I have had an abortion (take-out belly)”. The response to each of these lists was a single number – the number of items that a given participant had experienced. For the purposes of this paper, as opposed to the double list experiment described above, we only used data from List A as the methods described below were designed for a single list only. For half of our sample, respondents received List A exactly as listed above, and the other half received List A with abortion added as a fourth item.

### Testing for a design effect

A design effect exists if the expected number of control items reported depends on whether or not the list includes the sensitive item. [13] The absence of a design effect is the first of two key assumptions required for valid estimation and inference using list experiment data. [13] As an initial diagnostic for a design effect, we first calculated the difference between the treatment and control groups in the proportions of participants with at least one positive response, and then repeated this calculation for two through the number of control items. [10] If all of these differences were positive, it would be unlikely that a design effect was present. [13] But if some or all of the differences were negative, it is possible that some individuals altered their response to control items based on the presence of abortion on the list. Via the R list package by Blair and Imai 2010, [15] we implemented a likelihood ratio test [13] for whether the observed pattern was due to a design effect.

### Multivariable regression

Of direct substantive interest to many investigators will be the potential dependence on covariates of a positive response to the sensitive item. In this analysis, intended to be an exercise to demonstrate the application of these multivariable estimators, we examined whether a lifetime history of abortion depends on age and education. We acknowledge that education may be influenced by the safety and timing of earlier abortions, and that this limits inferences from this analysis. The primary objective of the analysis presented here, therefore, is to demonstrate the method of multivariable analysis, rather than to draw specific inferences from the results. To assess these questions, we implemented both nonlinear least squares and maximum likelihood estimators, both developed by Imai in 2011 [14]. Each of these approaches has distinct strengths and shortcomings.

The non-linear least squares (NLS) implements the analysis in two steps [14]. The first is to model the number of control items reported as a function of covariates, using data for the control group only. Then in the second step, the parameter estimates from this model are used in modeling the response to the sensitive item (abortion) in the treatment group, given the response to the control items and covariates [14]. A special case of the NLS occurs when one assumes that the two sub-models (for control and sensitive items respectively) are both linear. Under those assumptions, the NLS simplifies to a linear model with interactions between treatment and covariates [12, 14]. This model unfortunately does not constrain fitted values to the admissible range, and also requires use of methods that accommodate between-group differences in residual variance.

The second method, the maximum likelihood (ML) estimator, was developed to take fuller advantage of all of the information about the joint distribution of responses to the sensitive and control items [14]. This method uses maximum likelihood to estimate the parameters for two separate binomial models: the first for the probability of a positive response to the sensitive item (abortion), given covariates; and the second for the number of affirmative responses to the control list, given the response to the sensitive item and covariates. The complicated resulting likelihood is maximized using the expectation-maximization (EM) algorithm, treating the response to the sensitive item as partially missing data [13, 14]. We considered constrained and unconstrained versions of the ML estimator. The constrained version increases efficiency by forcing the parameters of the model for the number of positive control item responses to be the same in the treated and control groups [13]. All four of these estimators are implemented in the same “list” package in R used for assessing potential design effects.

In our application, we included age (in units of five years), and education, as a factor variable with four levels: no education (reference level), some or all elementary school, some or all of high school, and some or all of college. Seventy-nine women (2.4% of study sample) were excluded from analyses due to missing data on age (two individuals) and education (an additional 77 individuals).

All annotated R code is presented in Additional file 1.

## Results

Overall, 3464 women were approached, and 3291 women (95%) gave verbal informed consent to participate in the list experiment on abortion in Liberia. Women were 30 years old, on average, with a mean of three children, and most were in a committed relationship (Table 1). Several characteristics varied by urban versus rural residence, including parity and religion. Details of the sample have been reported elsewhere [6].

**Table 1:** Demographic characteristics of study sample, overall and by treatment assignment

Characteristic	Overall	List without abortion	List with abortion
	(*n* = 3285)	(*n* = 1676)	(*n* = 1609)
Means, ±SD			
Age, in years	30 ± 10	30 ± 10	30 ± 10
Parity	4 ± 2	4 ± 2	4 ± 2
Persons living in household	7 ± 4	7 ± 4	7 ± 4
Monthly household income, USD	$59 ± 388	$47 ± 105	$71 ± 543
Proportions, %			
Religion, %			
Muslim	28	26	29
Christian	72	73	70
Other	1	1	1
Education, %			
None	39	38	39
Some or all elementary	36	36	36
Some or all high school	21	20	21
Community college or university	4	5	3
Marital Status, %			
Single	26	26	26
Living with partner	35	33	36
Married	32	33	31
Divorced/separated	4	4	4
Widowed	4	4	4

### Test for design effect

Table 2 shows our initial diagnostic for design effects. The treatment-control difference in the proportions with at least one positive response is slightly negative, consistent with a design effect; however, the other four differences (for zero, two, three, and four items reported) are positive. The likelihood ratio test for the design effect is not statistically significant (*p* = 0.18). We conclude that there is no clear statistical evidence for a design effect in the Liberian list experiment data. In other words, because only one of the five differences was negative, and quite small in magnitude, there is no statistical evidence that respondents altered their responses to the control items based on whether or not abortion was added to the list.

**Table 2:** Results for test of no design effect assumption. Table contains estimates of the population proportion reporting each number of items, and at least each number of items, by treatment group

	Number of list items reported				
	0	1	2	3	4
Treatment list	0.040	0.278	0.446	0.168	0.068
Proportion at least	1.000	0.960	0.682	0.237	0.068
Control list	0.031	0.376	0.486	0.107	0.000
Proportion at least	1.000	0.969	0.593	0.107	0.000
Row 2 – Row 4	0.000	−0.009	0.089	0.130	0.068

A negative proportion in the bottom row suggests that the proportion reporting at least j items in the treatment group is less than the proportion reporting at least j items in the control group (Pr(Y > =j | *T* = 1) - Pr(Y > =j|*T* = 0) for j = 1,…, J). (J = 3, number of control item), and could be consistent with evidence for a design effect

### Multivariable regression outcomes

We present results from four models: linear least squares, non-linear least squares, constrained maximum likelihood, and unconstrained maximum likelihood (Table 3). Results from a likelihood ratio test comparing the two maximum likelihood models indicated that covariate effects on the number of positive control items were modified by the presence of the sensitive item (*p* = 0.02). All models assessed the relationship between age, education, and the sensitive item (abortion). Across all four models, results generally suggested an inverse association between higher educational attainment and abortion. For women with a high school education, this association was statistically significant at the alpha = 0.05 level in three of the four models. Women who had completed some or all of high school were only approximately one-third as likely to report ever having had an abortion as compared to women with no education (adjusted odds ratio [aOR]: 0.32–0.42, across models). No clear association between age and abortion, adjusting for education, was apparent. In three out of four control models, age was positively associated with report of control items, after accounting for education.

**Table 3:**
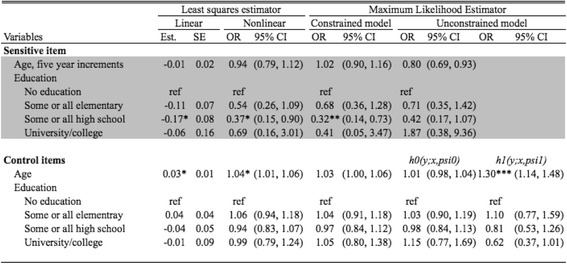
Estimated coefficients and odds ratios from the list experiment regression models where the sensitive item is whether or not the participant has had an abortion in her lifetime.

The coefficients of interest are age and education (highlighted in grey). Standard errors and 95% confidence intervals are listed for the linear and non-linear models, respectively

**Fig. 2:**
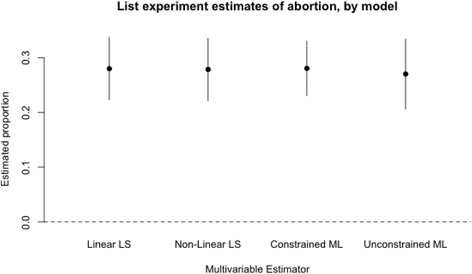
Estimated proportion of Liberian women who have had an abortion generated from each of four models, all adjusted for age and education. The solid circle represents the point estimate for the population proportion, adjusted for age education status, and the solid lines indicate the 95% confidence intervals

Standard errors for coefficient values are smallest in the linear least squares model, and comparable across the other three models. The precision of adjusted estimates of lifetime prevalence of abortion based on each of the four models is shown in Fig. 2. The constrained maximum-likelihood model is most precise.

## Discussion

We found no statistical evidence for a design effect in a list experiment conducted on abortion in Liberia, bolstering confidence in results from a method newly introduced to the health research field. Further, we demonstrated how multivariable analysis of list experiment results can be carried out with two multivariable estimators: a non-linear least squares estimator and a maximum likelihood estimator. Results from our multivariable analysis indicated that women with any education were less likely to respond affirmatively to the abortion list item than were women with no education, adjusting for age. These relationships were statistically significant for women with a high school education in three of the four models. We discuss the interpretation of these findings below.

In assessing evidence for a design effect, we found that one of the joint population proportions was slightly negative. This could indicate evidence that the presence of abortion on the treatment list affected the number of control items reported. However, it could instead be caused by chance, or could be due to a lack of exchangeability between the treatment and control groups [10]. In running the statistical test proposed by Blair and Imai 2012, [13] we found no evidence to reject the null hypothesis of no design effect. However, it is important to note that this test could potentially miss a design effect if some of the effect is positive and some of the effect is negative, such that the biases cancel each other out [13]. However, we think this pattern is unlikely in this instance given the nature of the non-sensitive items. It is also possible that a design effect may be present in certain strata of the sample, although not overall. However, given the lack of an a priori hypothesis about if and why a design effect may occur in certain subgroups, and given that estimates were reported for the sample overall, we only tested for a design effect on the full sample. In the instances in which list experiments return negative or nonsensical results, a design effect may be present. Implementation of the design effect test in future work can advance our understanding of the list experiment as a tool for family planning research, and identify contexts in which it works well, and others in which it does not.

In a previously published analysis, list experiment estimates of abortion were calculated for individual age categories in the Liberian sample. Estimates indicated that the percentage of women reporting abortion increased with age, as one would expect [6]. The multivariable analysis presented in this paper extended that research, and explored the association between age *and* education with report of abortion. After accounting for education, however, age was no longer associated with history of abortion. This finding is difficult to interpret. Liberia’s history of civil war undoubtedly influenced older women’s educational experiences, and also influenced the age structure of the population, but we do not feel confident speculating on the extent to which these factors may have influenced the relationship between age and history of abortion.

The finding that education is negatively associated with history of abortion after accounting for age is also difficult to interpret given the potential relationship between abortions that happen earlier in life and educational attainment. One possible interpretation could be that women with more education are better informed about contraception and less likely to have an unwanted pregnancy in the first place, thus reducing their likelihood of having an abortion. This interpretation is supported by prior work elsewhere in West Africa. For instance, one study relying on Demographic and Health Survey (DHS) data in Ghana found that women with a primary or secondary level of education were 1.5 times as likely to use contraception as compared to women with no education [16]. Results from another study, also relying on DHS data from a number of West African countries, similarly found that increased educational attainment and positive community norms toward women’s education were associated with increased likelihood of using modern contraception [17]. We know that increased use of contraception reduces the risk of unwanted pregnancy, which reduces the number of induced abortions [18].

Alternatively, due to legal restrictions on abortion in Liberia, it could be that women who have abortions in Liberia tend to have unsafe abortions with high morbidity (if not mortality) or with pervasive social stigma repercussions, which prevent them from continuing with their education. A study in Mexico using state-level population data from 32 federal states documented an inverse association between women’s educational attainment and abortion-related mortality [19]. While this could suggest, as described above, that women with more education are less likely to have abortions, and therefore to die from abortion, this association could also potentially unfold in the reverse direction: lower abortion-related mortality could lead to higher female educational attainment [19]. But without knowing when women had their abortions relative to their schooling, how safe those abortions were, and how this varied throughout our study sample, we cannot determine the direction of the association we observe in the multivariable results. Although information on educational history is of course non-sensitive, this list experiment did not elicit the required information on the timing and safety of abortions.

Consequently, investigators intent on using the list experiment to measure abortion should carefully consider the limitations of asking about lifetime history of abortion (or any sensitive health experience). Doing so will limit the utility of multivariable analysis of any resulting data. Asking instead about a more specific period of time, such as the past five to 10 years, would allow for the estimation of abortion incidence, and would limit reverse-causation bias in assessing multivariable relationships with participant demographic characteristics.

In terms of the specific estimators proposed, each has particular strengths and weaknesses. An advantage of the NLS estimator is that when the conditional mean functions are correctly specified, it is consistent [14]. A weakness, however, is that the linear form of the NLS estimator does not constrain fitted values to be between zero and the total number of possible items, and both the linear and non-linear forms do not make full use of the information on the joint distribution of control and sensitive item in the population, rendering it less statistically efficient than it could be [13].

The maximum likelihood estimator, in comparison, is more efficient because it uses the full information about the joint distribution of responses to sensitive and non-sensitive items by treatment status, and it is more amenable to use with hierarchical data, when that is of interest [14].

Investigators may question which approach is better on average. A 2011 simulation study compared the NLS and ML estimators in terms of bias, root mean square error (RMSE), and confidence interval coverage [14]. In estimating the unadjusted population proportion with the sensitive item, both performed equally well with regard to bias and coverage, although the ML estimate had smaller RMSE [14]. When looking at multivariable adjusted estimates, the ML estimator produced estimates with less bias and greater precision in small to moderate samples. However, performance was similar at sample sizes greater than ~3000 [14]. The overall estimate of the population proportion of women with history of abortion was most precise in the constrained ML model – consistent with results presented elsewhere [14].

## Conclusion

This paper aims to introduce several important analytical tools to researchers interested in employing the list experiment to measure abortion (or other sensitive public health events or behaviors) and to provide a worked example. The methods presented here are clearly explicated in the political science literature, [13, 14] but are new to a public health audience. The design effect test we discuss is crucial to assessing the validity of list experiment results, and may prove useful in interpreting results that do not match expectations. This paper demonstrates that multivariable analyses, as well as statistical testing of a key design assumption, are possible with list experiment data on abortion, although with important limitations when considering cumulative lifetime assessment. We hope that the example presented here will facilitate the use of list experiments by others in the field, and thereby expand the suite of tools available for measurement of elusive public health populations.

## Additional file

Additional file 1: Annotated R Code. (DOCX 166 kb)

## Abbreviations

EA: Enumeration area
EM: Expectation maximization algorithm
ML: Maximum likelihood estimator
NLS: Non-linear least squares estimator
RMSE: Root mean square error
