# Supplementary material for: Multivariable regression analysis of list experiment data on abortion: results from a large, randomly-selected population based study in Liberia
Source: Popul Health Metr. 2017 Dec 21;15:40. doi: 10.1186/s12963-017-0157-x (PMC5740939; doi:10.1186/s12963-017-0157-x)
Supplement: Additional file 1: — Annotated R Code. (DOCX 166 kb) [file 12963_2017_157_MOESM1_ESM.docx]

**Appendix A: Annotated R Code**

# Install list package from Blair & Imai

install.packages(‘list’)

# Load package

library(‘list)

# Define function for printing odds ratios for ML and NLS results

print.ors<-function(fit,ndec=2) {

or<-round(exp(fit$par.treat),ndec)

lb<-round(exp(fit$par.treat+qnorm(.025)*fit$se.treat),ndec)

ub<-round(exp(fit$par.treat+qnorm(.975)*fit$se.treat),ndec)

p.value<-round(2*pnorm(abs(fit$par.treat/fit$se.treat),lower.tail=F),4)

cat("\nSensitive item:\n")

print(cbind(or,lb,ub,p.value)[-1,])

if(!is.null(fit$par.control)) {

or<-round(exp(fit$par.control),ndec)

lb<-round(exp(fit$par.control+qnorm(.025)*fit$se.control),ndec)

ub<-round(exp(fit$par.control+qnorm(.975)*fit$se.control),ndec)

p.value<-round(2*pnorm(abs(fit$par.control/fit$se.control),lower.tail=F),4)

cat("\nControl item count:\n")

print(cbind(or,lb,ub,p.value)[-1,])

}

else {

or<-round(exp(fit$par.control.psi0),ndec)

lb<-round(exp(fit$par.control.psi0+qnorm(.025)*fit$se.control.psi0),ndec)

ub<-round(exp(fit$par.control.psi0+qnorm(.975)*fit$se.control.psi0),ndec)

p.value<-round(2*pnorm(abs(fit$par.control.psi0/fit$se.control.psi0),lower.tail=F),4)

cat("\nControl item count, negative sensitive item response:\n")

print(cbind(or,lb,ub,p.value)[-1,])

or<-round(exp(fit$par.control.psi1),ndec)

lb<-round(exp(fit$par.control.psi1+qnorm(.025)*fit$se.control.psi1),ndec)

ub<-round(exp(fit$par.control.psi1+qnorm(.975)*fit$se.control.psi1),ndec)

p.value<-round(2*pnorm(abs(fit$par.control.psi1/fit$se.control.psi1),lower.tail=F),4)

cat("\nControl item count, positive sensitive item response:\n")

print(cbind(or,lb,ub,p.value)[-1,])

}

}

# Define function for likelihood ratio test for comparing unconstrained and constrained ML fits

lrtest<-function(constrained.fit, unconstrained.fit) {

X2<-2*abs(constrained.fit$llik-unconstrained.fit$llik)

df<-length(unconstrained.fit$coef.names)

lrp<-pchisq(X2,df,lower.tail=F)

cat("\nLR test comparing constrained (ll=", constrained.fit$llik,

") and unconstrained (ll=", unconstrained.fit$llik, ") ML models:\n X2=", X2, ", df=", df, ", P=", round(lrp,4), "\n", sep="")

}

# Load data for List Experiment analysis

load("/Users/Username/File/l1.Rdata")

# Test No Design Effect Assumption:

# l1 = dataset

# list1= list items reported by respondent

# tx1 = binary variable denoting whether individual received treatment or control list

# J = number of control list items

designtest<-ict.test(l1$list1, l1$tx1,J=3, gms = TRUE)

print(designtest)

# Generate estimate of population proportion based on difference in means

diff.in.means.results<-ictreg(list1~1, data=l1,treat="tx1", J=3,method='lm')

summary(diff.in.means.results)

# Linear Least Squares Estimator

lm.results<-ictreg(list1 ~ var1 + var2, data=l1, treat="tx1",J=3, method="lm")

summary(lm.results)

# Non-Linear Least Squares Estimator

nls.results<-ictreg(list1 ~ var1 + var2, data=l1, treat="tx1",J=3, method="nls")

summary(nls.results)

print.ors(nls.results)

# Constrained Maximum Likelihood Estimator

ml.cons.results<-ictreg(list1 ~ var1 + var2, data=l1, treat="tx1",J=3, method="ml", overdispersed=FALSE,constrained= TRUE)

summary(ml.cons.results)

print.ors(ml.cons.results)

# Unconstrained Maximum Likelihood Estimator

ml.uncons.results<-ictreg(list1 ~ var1 + var2, data=l1, treat="tx1",J=3, method="ml", overdispersed=FALSE,constrained=FALSE)

summary(ml.uncons.results)

print.ors(ml.uncons.results)

# Likelihood ratio test for comparison of constrained & unconstrained ML models

summary(ml.cons.results)

summary(ml.uncons.results)

lrtest(ml.cons.results,ml.uncons.results)
